# Biopolymer-protected graphene-Fe_3_O_4_ nanocomposite based wearable microneedle sensor: toward real-time continuous monitoring of dopamine†

**DOI:** 10.1039/d4ra00110a

**Published:** 2024-02-27

**Authors:** Keerthanaa M. R., Lakshmi R. Panicker, Roger Narayan, Yugender Goud Kotagiri

**Affiliations:** a Department of Chemistry, Indian Institute of Technology Palakkad Palakkad Kerala 678 557 India yugenderkotagiri@iitpkd.ac.in; b Department of Biomedical Engineering, NC State University Raleigh NC 27695 USA

## Abstract

Neurological disorders can occur in the human body as a result of nano-level variations in the neurotransmitter levels. Patients affected by neuropsychiatric disorders, that are chronic require continuous monitoring of these neurotransmitter levels for effective disease management. The current work focus on developing a highly sensitive and personalized sensor for continuous monitoring of dopamine. Here we propose a wearable microneedle-based electrochemical sensor, to continuously monitor dopamine in interstitial fluid (ISF). A chitosan-protected hybrid nanomaterial Fe_3_O_4_–GO composite has been used as a chemical recognition element protected by Nafion antifouling coating layer. The morphological and physiochemical characterizations of the nanocomposite were carried out with XRD, XPS, FESEM, EDAX and FT-IR. The principle of the developed sensor relies on orthogonal detection of dopamine with square wave voltammetry and chronoamperometric techniques. The microneedle sensor array exhibited an attractive analytical performance toward detecting dopamine in phosphate buffer and artificial ISF. The limit of detection (LOD) of the developed sensor was observed to be low, 90 nM in square wave voltammetry and 0.6 μM in chronoamperometric analysis. The practical applicability of the microneedle sensor array has been demonstrated on a skin-mimicking phantom gel model. The microneedle sensor also exhibited good long-term storage stability, reproducibility, and sensitivity. All of these promising results suggest that the proposed microneedle sensor array could be reliable for the continuous monitoring of dopamine.

## Abstract 2

Neurological disorders can occur in the human body as a result of nano-level variations in the neurotransmitter levels.

## Graphical abstract

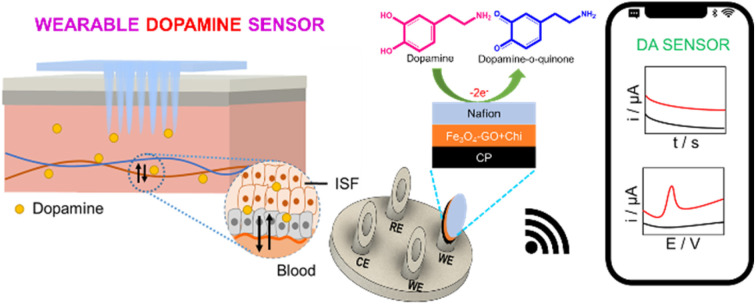

## 1. Introduction

The neurological disorders caused due to abnormal levels of neurotransmitters were found to be the leading cause of long-term disabilities, and the second leading cause in terms of mortality worldwide.^1^ In India, neurological disorders are estimated to increase by 23% between 2013 and 2025.^2^ It was also reported that, as a result of the recent COVID-19 pandemic, cognitive, psychiatric and neurological functions are being disturbed in patients affected by SARS-CoV-2.^3^ Therefore, the detection and monitoring of neurotransmitters are significant in this regard. A neurotransmitter is a chemical messenger whose functions are directly linked to the central nervous system and can influence various physiological and psychological activities.^4^ Neurotransmitters are released as a response to electrical stimulation and act on their respective receptors, initiating simple to complex responses.^5^ An umbrella term to denote the disorders due to catabolism, synthesis and mechanism of neurotransmitter discrepancy or imbalance is ‘Neurotransmitter disease’. When the signal transduction between the neurons is affected due to abnormalities in neurotransmitter levels, diseases like Segawa disease, Alzheimer's, autism, autism spectrum, Parkinson's, bipolar disorder, epilepsy and dementia arise. These abnormalities in neurotransmitter levels can occur as a result of caffeine intake, genetic predisposition, alcohol and drugs, poor diet, or other environmental factors.^6^ These neurotransmitters need to be detected and continuously monitored for effective management of neurological diseases. Dopamine, serotonin, acetylcholine and glutamate are common neurotransmitters responsible for various neurological disorders. Dopamine, being associated with several chronic neurological disorders, requires detection and continuous monitoring.

Dopamine (DA) belongs to the catecholamine class of monoamine neurotransmitters.^7^ It plays an essential role in the human central nervous system in the brain involving various brain functions like memory, motor control, motivation, arousal, sexual behaviour, reward and pleasure, emotions and mood, behavioural responses which include feeling, addiction and transduction responses, also influencing renal, hormonal and cardiovascular systems.^7–10^ Abnormal levels and lack of dopamine are associated with several psychiatric disorders, including Alzheimer's, drug addiction, aprosexia, Parkinson's, schizophrenia, Huntington's disease, and attention deficit hyperactivity disorder (ADHD).^7,8,11^ Conventional laboratory-based healthcare technologies demand patients to reach the hospitals causing detrimental effects from lengthy diagnosis time to delayed treatment.^12^ The increasing need for diagnostic instruments that include improved analytical attributes such as sensitivity, selectivity, and short response time has prompted researchers to focus their efforts on the advancement of point-of-care (POC) devices.^13^

POC tests offer a high level of convenience for the continuous monitoring of specific analytes or for repeating follow-up tests aimed at tracking the course of treatment.^14^ Various POC devices are being developed for the detection of dopamine owing to its clinical importance. Some of the recently reported methods include graphene-based flexible biosensors, paper microchip capillary electrophoresis, fully-integrated and handheld electrochemiluminescence device.^15–17^ Highly stretchable POC sensors using sweat and ISF as biofluids for monitoring glucose has also been developed.^18,19^ Point-of-care diagnostics are fast and patient-centered, while conventional disease diagnostics are time-consuming, require skilled personnel in labs and hospitals, and are expensive, instead, wearable sensors capture data while patients continue with their regular routines.^20^ Wireless POC devices or wearable sensors can continuously monitor biologically relevant parameters, metabolites, and bio-molecules, which can help treat morbid diseases like Alzheimer's.^21^ In the past decade, the development of wearable electrochemical sensors for the detection of various bio-fluids has provided new opportunities for the management of clinical data of patients with chronic illnesses like Parkinson's, and diabatic.^22^

Wearable sensors and portable devices together have the potential to put forward a shift in health care from traditional methods to continuous monitoring of patients in the comfort of their home.^23^ Fluids like saliva, sweat, tears, and subcutaneous ISF, from the human body, are capable of real-time monitoring of proteins, sweat metabolites, warfare agents, toxic heavy metals, electrolytes, environmental gases, drug monitoring, *etc.*^12,24^ ISF has the most remarkable ability to replace the current gold standard diagnosis based on blood. ISF is formed through the transcapillary filtration of blood and cells and tissues of the body and cells are surrounded by ISF. Owing to its close contact with blood, itis found to have a similar composition to that of blood, including biomarker concentrations. ISF has various advantages over blood samples, due to a lack of coagulating factors, this can be sampled in a minimally invasive. Minimally invasive microneedle-based electrochemical sensors are capable of on-the-tip sensing and offer the potential for integration with miniaturized devices.^25^ Recently electrochemical wearable sensors have been developed for the application of continuous monitoring of biomarkers like, glucose,^26^ lactate,^27^ alcohol^28^*etc.*, and drugs such as levodopa,^29^ phenoxymethylpenicillin^30^ and apomorphine.^31^ Similar to the biomarker levels, catecholamine levels vary upon different factors (even with acute stress), which needs to be continuously monitored.^32^ Hence the developed biosensor must be sensitive in nature. Nanomaterial-based electrochemical sensors exhibit notable electrocatalytic enhancement and electronic conductivity proving to be efficient for behaving as a chemical recognition element.^33^

Carbon-based nanomaterials have attracted attention through their high conductivity, mechanical strength, large surface area, and faster mobility of charges.^34^ Oxidation of graphite produces graphene oxide, a derivative of graphene that contains different functional groups. The functional groups, that are present on the surface of GO play a crucial role in controlling its optical transparency, electrical conductivity, and thermal conductance.^35^ Transition metal oxide-based materials are commonly used in the fabrication of biosensors owing to their structural stability and catalytic activity.^36^ Fe_3_O_4_, a transition metal-oxide-based nanomaterial has properties like biocompatibility, structural stability non-toxic and inexpensive.^37^ Moreover, Fe_3_O_4_–GO nanocomposite was prepared by the chemical coprecipitation method, which is cheap and environmentally friendly. The biopolymer used here is chitosan, it is a naturally available polysaccharide extensively used in biomaterial applications. Properties like biocompatibility, biodegradability, low toxicity, and bioactivity can be attained with this inexpensive biopolymer.^38,39^ The Fe_3_O_4_–GO nanoparticles are uniformly dispersed in 0.5% (W/V) chitosan.

Previous attempts have been made for dopamine detection and monitoring few of them are, molecularly imprinted electrochemical sensors for multiplexed metabolites detection,^40^ Ryu *et al.*, developed a swellable hydrogel-based electrochemical sensor for dopamine detection in ISF,^41^ and Antiochia *et al.*, proposed a nanoporous gold-based sensor for catecholamine detection.^42^ In this article, a microneedle-based biosensor has been developed for the continuous monitoring of neurotransmitter dopamine. The carbon paste-filled hollow microneedles were surface-modified with Fe_3_O_4_–GO/chi nanocomposite. Further Square Wave Voltammetry (SWV) and chronoamperometry (CA) techniques were employed for the detection of dopamine. Characterizations of working electrode surfaces were carried out with electroanalytical techniques and surface morphological studies. In phosphate buffer solution (PBS) an orthogonal sensing of DA was done and the ability of the sensor in real-time monitoring was evaluated in artificial ISF medium and phantom skin mimicking gel. This wearable microneedle biosensor exhibited a desirable sensitivity, selectivity, and long-term stability for the continuous monitoring of dopamine.

## 2. Materials and methods

### 2.1. Chemicals

Iron(iii) chloride hexahydrate (FeCl_3_·6H_2_O), iron(ii) chloride tetrahydrate (FeCl_2_·4H_2_O), graphite nanopowder (type-1), paraffin liquid oil (extrapure), potassium permanganate (KMnO_4_), ammonium hydroxide (NH_4_OH), concentrated sulphuric acid (H_2_SO_4_), hydrogen peroxide (H_2_O_2_), sodium nitrate (NaNO_3_), potassium chloride (KCl), potassium ferricyanide ([K_3_Fe(CN)_6_]) extrapure 98%, agarose (low EEO), CaCl_2_ fused pure (90–95%), sucrose, sodium borohydride powder (NaBH_4_), magnesium sulphate dried extrapure (MgSO_4_·*x*H_2_O), sodium phosphate monobasic dihydrate extrapure (NaH_2_PO_4_·2H_2_O) and sodium bicarbonate extrapure (NaHCO_3_) were purchased from SRL chemicals. Dopamine hydrochloride, uric acid, acetic acid and serotonin hydrochloride were purchased from Sigma-Aldrich. Chitosan (0.5% in 0.5% acetic acid) obtained from Tokyo Chemical Industry (TCI Chemicals). Glucose (d-glucose anhydrous) was purchased from Nice Chemicals. All the mentioned chemicals were used for analysis without any further purifications.

### 2.2. Instrumentation

The electrochemical tests were conducted utilizing the PalmSens EmStat3 handheld potentiostat, which was operated by PS Trace software version 5.9. FT-IR analyses were carried out in Schimadzu Scientific Instrument. The X-ray diffraction (XRD) spectra were obtained using a Smart Lab XR apparatus manufactured by Rigaku Corporation. The equipment utilized copper K-alpha radiation with a wavelength of 0.154 nm and was operated at a voltage of 45 kV. The experiment involved collecting data within a 2*θ* range of 10–90°. The data was obtained by taking measurements at regular intervals of 0.02° and each measurement was performed for a duration of 0.3 s. This approach allowed for the acquisition of diffraction patterns with a high level of detail and precision. The morphological study of the composite material was conducted using Field Emission Scanning Electron Microscopy (FE-SEM) and Energy Dispersive X-ray analysis (EDX) with the aid of a Gemini SEM 300 instrument (manufactured by Carl Zeiss). An accelerating voltage of 15 kV was applied during the analysis process.

### 2.3. Preparation of graphene oxide

The modified Hummers' method was used to synthesize graphene oxide (GO) from graphite powder.^43^ The process of preparation is as follows: pre-oxidation of 1.5 g of graphite powder was performed using 20 ml of concentrated H_2_SO_4_ and 0.75 g of NaNO_3_. The mixture was then swirled for at least 4 hours while being kept cool. After that, 4.5 g of KMnO_4_ was added slowly and the mixture was mixed thoroughly for a further hour while remaining cold. The solution was heated to 50 °C and stirred for 4 hours. Then, 50 ml of distilled water was added, and the temperature was increased from 50 °C to 90 °C while being kept constant for 1 hour. The solution mixture was added with 100 ml of double-distilled water and 12.5 ml of 30% H_2_O_2_, which changed the colour of the solution mixture to a brownish hue. This mixture was then continuously stirred for an hour. The GO solid materials were kept drying for future use after being centrifuged and repeatedly cleaned with 5% HCl and double-distilled water. It was dried at 80 °C for one hour and kept for further use.

### 2.4. Synthesis of Fe_3_O_4_–GO–chitosan nanocomposite

Initially, 0.2 g FeCl_3_·6H_2_O and 0.4 g FeCl_2_·4H_2_O were taken and dissolved in 50 ml de-ionized water. Then 0.5 g GO was added and ultrasonicated for 30 min pH of this solution was controlled to 10 with the pH meter using 25% ammonium solution under vigorous stirring for 5 h. The supernatant solution was removed and the black precipitate was washed with water and dried at 80 °C for 24 h. Fe_3_O_4_–GO nanocomposite was obtained by grinding this precipitate with mortar.^44^ Further, the nanocomposite was dispersed in 0.5% (W/V) of chitosan solution and sonicated for 30 minutes. This makes the synthesized nanocomposite biopolymer protected. The mentioned synthesis of nanoparticles is schematically represented in Fig. S1 in ESI.†

### 2.5. Fabrication of modified microneedle

Fusion 360 software designed hollow microneedle arrays. Stereolithography 3D printing created the microneedle's polymethylmethacrylate (PMMA) substrate. Pyramidal needles have circularbases. The microneedle sensor has 1 × 4 pyramidal hollow needles with 500 μm diameters and 1500 μm heights.^45^

The fabricated microneedle was subjected to stress analysis upon insertion using Fusion 360 software. Skin penetration force, 3.16 MPa was applied on the microneedle model using simulation. Stress of 8.045 MPa, was found to be developed on the tip of the microneedle, this is less than the yield strength of PMMA. Through this analysis, it can be concluded that the microneedle will not break upon insertion through the skin. Fig. S2† shows the result of stress analysis in Fusion 360 software.

On a circular microneedle patch, there are four hollow microneedles distributed. The diameter of each hollow microneedle is 0.5 mm which is placed at a 10 mm spacing^46^ These were split into two working electrodes (WE), one counter electrode (CE), and one reference electrode (RE). A 5 mm long Ag wire is fitted into a microneedle space which will be acting as a RE. The hollow spaces of working and counter microneedles are filled with CP, composed of 65 weight percent graphite powder and 35 weight percent paraffin oil/mineral oil, to make them conductive. Electrical connections were made from the backside with stainless copper wires, placed over the sealed backside coated with silver epoxy ink and made to cure at 80 °C for an hour. Extra CP over the microneedle tip surface was removed cautiously with the help of a surgical blade and polished. The working electrode is modified with the Fe_3_O_4_–GO–chi composite for further electrochemical studies. Nafion coating was finally added as a protective layer over the working electrode. Nafion is a widely used ion-exchange polymer membrane and found to play a crucial role in immobilizing electroactive cations over the electrode surface.^47,48^

### 2.6. Electrochemical analysis

The electrochemical tests were conducted by the PalmSens EmStat3 handheld potentiostat, which was operated by the PS Trace software version 5.9. The optimization of the synthesized material was conducted through the cyclic voltammetry measurements in a 10 mM ferricyanide solution. This involved applying a potential range of −0.2 to 0.9 V, with a scan rate of 100 mV s^−1^. Subsequently, the electroanalytical characteristics of the microneedle sensor that was constructed were evaluated in a 0.1 M phosphate-buffered saline (PBS) solution at a pH of 7, as well as in an artificial ISF solution. This evaluation was conducted with square wave voltammetry and amperometry transduction techniques. In square wave voltammetry, a potential range ranging from −0.2 to 1 V was applied, employing optimum settings including a frequency of 10 Hz, a step potential of 10 mV, and an amplitude of 50 mV. Chronoamperometric measurements were conducted using an applied potential of 0.3 V relative to Ag/AgCl for a duration of 60 seconds. The stability of the sensor was assessed through chronoamperometry over an extended duration. The selectivity of the sensor was evaluated by employing the chronoamperometry technique in the presence of various common interfering compounds. The efficacy of the microneedle sensor has been shown by its application in a gel-mimicking model.

### 2.7. Preparation of artificial ISF

The electroanalytical performance of the microneedle sensor with the modified electrode was studied in artificial ISF. The microneedles are minimally invasive and can detect the biomarkers present in the ISF layer of skin. Coagulating agents are absent in ISF, whereas blood has a coagulating nature considering this property of ISF, this can act as biofluid for continuous monitoring of different neurotransmitters and therapeutic drugs. Artificial ISF was prepared following the literature.^49^ In short NaCl (0.0315 g), KCl (0.014 g), MgSO_4_ (0.0085 g), NaHCO_3_ (0.11 g), NaH_2_PO_4_ (0.013 g), glucose (0.03 g), sucrose (0.13 g) and CaCl_2_ (10%) were weighed and dissolved in 50 ml of 0.1 M PBS.

### 2.8. Phantom skin mimicking studies

Human skin was mimicked based on agarose phantom gel, based on previous microneedle-based work by K. Yugender Goud *et al.*^50^ Initially, 280 mg of agarose was dissolved in 20 ml of 0.1 M PBS and stirred at 120 °C until it was completely dissolved. Now, this homogeneous agarose solution was poured into a Petri dish and left to solidify. Four different concentrations of dopamine were made and then poured into Petri dishes containing gel. The analyte was allowed to diffuse for 60 min at room temperature. The microneedle sensor array with a modified electrode was subjected to phantom hydrogel experiments, where the needles pierced through the gel and were tested after allowing a 1 min contact of needle surface and gel followed by chronoamperometric detection of dopamine.

## 3. Results and discussion

### 3.1. Design and principle of microneedle sensor

**Fig. 1:**
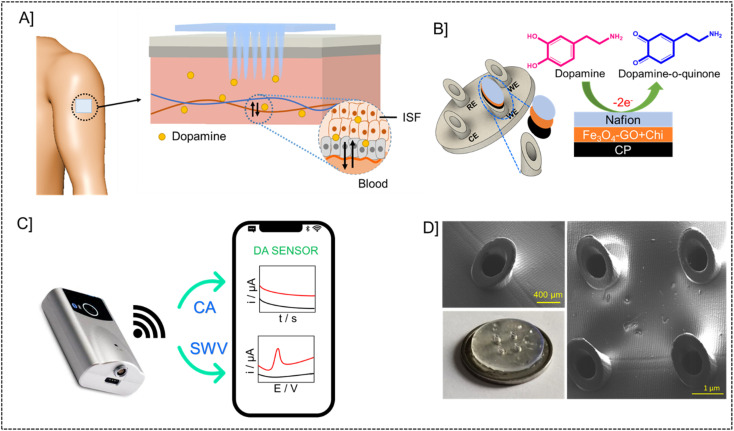
Electrochemical microneedle-based sensor for continuous monitoring of Dopamine. [A] Schematic representation of microneedle patch on the skin and microneedle array piercing through the skin. [B] Working electrode modifications with carbon paste, Fe_3_O_4_–GO–chitosan nanocomposite and Nafion. [C] Portable potentiostat device with a mobile phone for orthogonal detection of dopamine. [D] Real image in comparison with the size of a coin and FESEM images of empty microneedles.

The microneedle sensor orthogonally detects dopamine at the modified working electrode. The design of hollow microneedle arrays was accomplished by the utilization of multifunctional computer-aided fusion 360 software^45^ The substrate material for the microneedle was polymethylmethacrylate (PMMA), an FDA-approved polymer and hence can be used for building microneedle platform, which was fabricated utilizing the stereolithography 3D printing process. The microneedle patch on human skin is schematically represented in Fig. 1A. The microneedle dimensions are designed such that it is sharp and penetrate only up to the ISF thereby preventing nerves from damage. This enables the continuous monitoring of DA in ISF. Fig. 1B shows the modifications on the working electrode with carbon paste, Fe_3_O_4_–GO/chi, and Nafion. The modified working electrode acts as a transducer where dopamine is oxidized to dopamine-*o*-quinone since it is electroactive in nature. The Bluetooth enabled handheld potentiostat device along with a mobile phone that can be used for the monitoring of dopamine in ISF is represented in Fig. 1C. The FESEM images and real images of a microneedle (in comparison with a coin) used to develop this sensor are depicted in Fig. 1D. The needles exhibited a pyramidal morphology. The microneedle sensor is comprised of a set of 1 × 4 pyramidal hollow needles, each possessing a diameter of 500 μm and a height of 1500 μm. The inter-needle spacing measures 1 mm.

The microneedle sensor array carries four hollow spaces. Among them three were filled with carbon paste and the surface was polished. One will be acting as a counter electrode labeled as CE, while two others will be serving as working electrodes labeled as WE1 and WE2. Ag wire inserted through the remaining hollow space serves as a RE. Fe_3_O_4_–GO–chi nanocomposite was drop-casted over the working electrode surfaces. The modified and unmodified working electrodes were evaluated initially in 0.1 M PBS (pH-7), followed by artificial ISF, and finally in phantom skin-mimicking gel.

### 3.2. Structural and morphological characterization of Fe_3_O_4_–GO nanocomposite

#### 3.2.1. Structural characterizations

The structural characteristics of the nanocomposite were investigated with studies like FT-IR spectroscopy, XRD and XPS. The formation of the Fe_3_O_4_–GO composite was confirmed by interpreting the data obtained. The FT-IR results of GO shows functional group peaks around 2800–3000 cm^−1^, 1480–1520 cm^−1^, 1650–1700 cm^−1^, 1220 cm^−1^, 1000 cm^−1^ and 700 cm^−1^. In the case of Fe_3_O_4_–GO nanocomposite, the intensity of all the functional group peaks in GO was found to be less intense and a broad peak around 560–800 cm^−1^ was observed in addition to function group peaks (Fig. S3†). The broad peak (560–800 cm^−1^) is the characteristic peak of the Fe–O bond and through the FT-IR spectrum it can be observed that Fe_3_O_4_–GO is formed. The XRD of Fe_3_O_4_–GO nanocomposite and GO is given in Fig. S4† 2*θ* values of GO peaks were observed around 20° and 42.2°, the peak of graphite was not visible at 26° and thus formation of GO can be confirmed. Fe_3_O_4_–GO nanocomposite shows a GO peak shift from 20° to 23° and is less intense. The peaks are observed at 35.52°, 43.36°, 57.30° and 62.78° that can be indexed to (3 1 1), (4 0 0), (5 1 1) and (4 4 0).

**Fig. 2:**
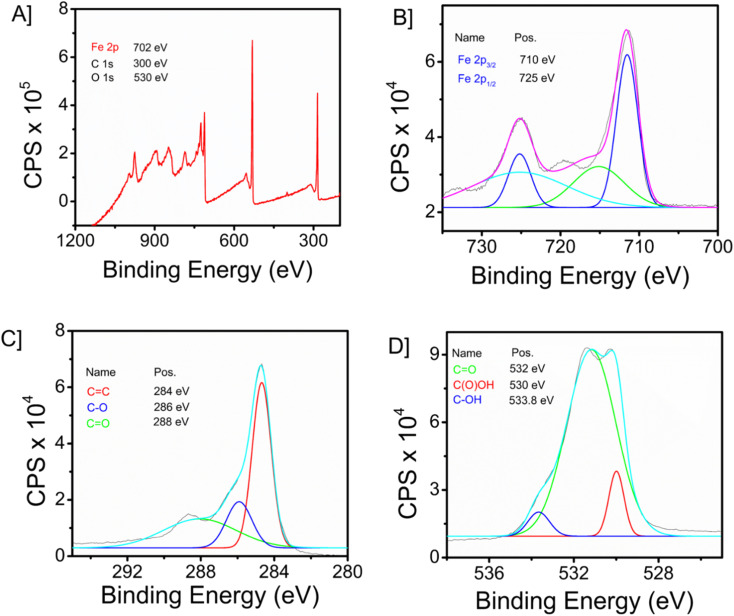
[A] XPS survey spectrum of Fe_3_O_4_–GO nanocomposite. [B] High-resolution XPS of iron, [C] oxygen, and, [D] carbon.

The X-ray photoelectron spectroscopy (XPS) technique is widely recognized as a robust analytical instrument that enables the investigation of the elemental composition of a given material. Fig. 2A depicts the survey spectrum of the synthesized composite material. The survey spectrum displays prominent peaks at 702, 300, and 530 eV, corresponding to the primary elements of iron (Fe 2p), carbon (C 1s), and oxygen (O 1s), respectively. Fig. 2B–D illustrate the high-resolution spectra of iron, oxygen, and carbon, respectively. In the instance of the Fe 2p high-resolution XPS spectra (Fig. 2B), two prominent peaks were identified at 725 eV (Fe 2p_1/2_), together with a peak at 710 eV (Fe 2p_3/2_). The C 1s spectra, as illustrated in Fig. 2C, exhibit three peaks. These peaks correspond to the graphite carbon main peak C=C (284 eV), C–C (286 eV), and the carbon present in C=O (288 eV). Fig. 2D depicts the representation of comparable high-resolution spectra of oxygen 1s, which exhibits three distinct peaks at 532 eV (C=O), 530 eV (C(O)OH) and 533.8 eV (C–OH). The prominent peak observed at 284.8 eV (C=C) in the C 1s spectrum signifies the presence of a highly graphitic structure in graphene oxide (GO).

#### 3.2.2. Morphological characterization

**Fig. 3:**
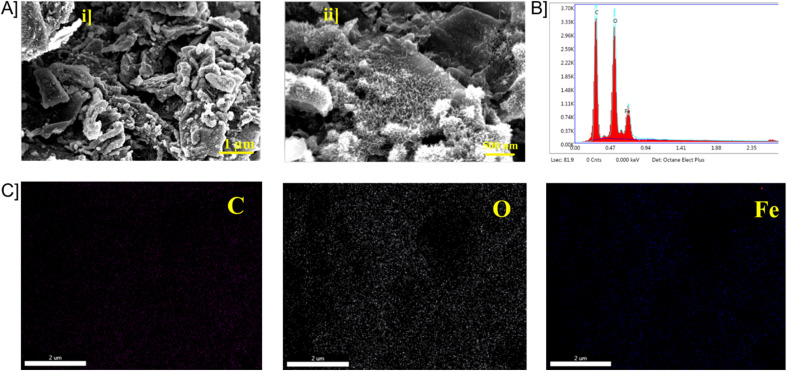
[A] FE-SEM images of Fe_3_O_4_–GO [B] EDX mapping of Fe_3_O_4_–GO and elemental mapping of carbon. [C] Elemental mapping of oxygen and iron.

The SEM images of Fe_3_O_4_–GO are shown in Fig. 3A in scales 1 μm and 500 nm. The morphology of GO was observed as flakes/thin layers and Fe_3_O_4_ nanoparticles are seen accumulated randomly over the GO surface. Aggregation of Fe_3_O_4_ nanoparticles is seen due to the strong magnetic attraction between Fe_3_O_4_ nanoparticles and the effect of surface energy. Magnetic interaction between Fe_3_O_4_ and GO causes Fe_3_O_4_ particles to deposit on the surface of GO. From EDAX elemental analysis, oxygen content was found to be 44%, followed by carbon having 40% and iron 14% (Fig. 3B). The ratio between carbon (C atom, 40%) and oxygen (O atom, 44%) was found to be approximately 1 : 1 indicating good composition of the nanocomposite. It can be inferred that only carbon (C atom, 40%), oxygen (O atom, 44%) and iron (Fe atom, 14%) are found in the hybrid-nanocomposite. Hence the nanocomposite does not contain any other elements and is of high purity.

### 3.3. Optimizations

Electrocatalytic transducing materials serve a significant role in assisting direct electrochemical voltammetry and amperometry-based sensors. In order to improve the catalytic transducing surface, it is necessary to optimize the system to enhance dopamine (DA) oxidation peaks. To develop a sensitive microneedle sensor, parameters like solvent or polymer in which the nanocomposite is dispersed and made as an ink and number for transducer layers. A good electrode surface requires uniform dispersion of nanocomposite and layer formation once drop-casted. Optimization was carried out in organic solvent (ethanol) and biopolymer (chitosan). Ethanol which is an organic solvent provided uniform dispersion of nanoparticles but the film formation over the electrode was not as expected owing to its volatile nature. The biopolymer chitosan gave a proper dispersion as well as good layer formation over the electrode surface. The current response of chitosan was higher compared to that of ethanol dispersed layer. Fig. S5† gives the current response of Fe_3_O_4_–GO in chitosan and ethanol recorded in cyclic voltammetry (10 mM Ferri solution). Different amounts of material were dispersed in the desired chitosan and CV responses were recorded. A higher current was obtained for 1 mg ml^−1^ dispersion of Fe_3_O_4_–GO. The optimized parameters were used for further studies. The frequency and amplitude of the square wave voltammetry are a few other parameters of significance. Initially, experiments were carried out with a frequency range of 10–50 Hz and the corresponding results were analyzed. A frequency of 10 Hz was found to be optimum and defined for the DA monitoring. With a frequency of 10 Hz, the influence of amplitude was studied at amplitudes ranging from 10 to 50 mV. Favourable results were obtained at 50 mV. Thus, subsequent SWV measurements were carried out with 10 Hz frequency and 50 mV amplitude.

### 3.4. Electrode–electrolyte interface characterizations

**Fig. 4:**
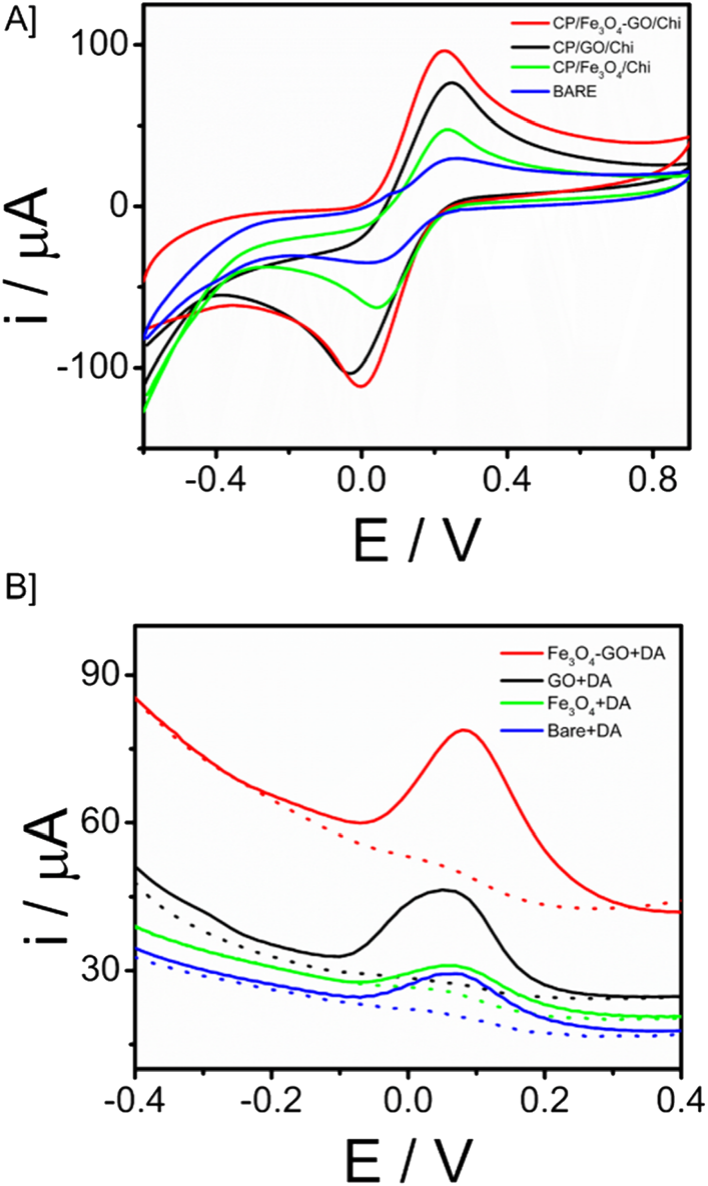
[A] CV responses of different modified electrodes recorded in 10 mM K_3_[Fe(CN)_6_] + 0.1 M KCl at an applied potential of −0.6 V to 0.9 V and scan rate of 100 mV s^−1^. [B] SWV comparison of CP/Fe_3_O_4_–GO/chi electrode with other modified and unmodified electrodes toward 30 μM dopamine.

In the electrode–electrolyte interface studies, cyclic voltametric analysis was done with different working electrode modifications cyclic voltammetry is a widely used electrochemical technique that is highly regarded for its effectiveness in studying the reduction and oxidation processes of molecular species. CP/Fe_3_O_4_–GO/chi, CP/Fe_3_O_4_/chi, CP/GO/chi and bare electrode (unmodified – CP) were tested in 10 mM Ferri (K_3_[Fe(CN)_6_] + 0.1 M KCl) solution with a scan rate of 100 mV s^−1^. The electrochemical behaviour of the Fe_3_O_4_–GO modified electrode is higher compared to that of other modified and bare electrodes. The current response of CP/Fe_3_O_4_–GO/chi electrode was observed as 67 μA compared to bare giving 30 μA, the current increase is more than twice the bare response. In cyclic voltametric response, a slight shift of anodic peak current towards negative potential is observed for the modified electrode CP/Fe_3_O_4_–GO/chi shown in Fig. 4A. Through this response, it exhibits superior electrochemical behaviour than other electrodes. The increased current response and potential shifts are clear indications of the conducting and catalytic properties of the transducer surface. Fig. S6A† is the cyclic voltametric response of CP/Fe_3_O_4_–GO/chi in different scan rates. An increasing scan rate from 10 to 100 mV s^−1^ in 10 mM K_3_[Fe(CN)_6_] + 0.1 M KCl, an increase in *i*_pa_ and cathodic peak current (*i*_pc_) was observed. A plot of *i*_pa_ and *i*_pc_*versus* square root of scan rate is linear (Fig. S6B†) depicting the diffusion-controlled process and surface-confined nature of the reaction.

Further studies were carried out in square wave voltammetry, in order to determine the ability of the CP/Fe_3_O_4_–GO/chi electrode to detect the neurotransmitter dopamine. Square-wave voltammetry is a differential technique of significant amplitude that involves the application of a waveform consisting of a symmetric square wave, overlaid on a base staircase potential, to the working electrode. Fig. 4B depicts the SWV responses of the modified electrode in comparison with different working electrodes toward the detection of 30 μM dopamine. It was observed that the CP/Fe_3_O_4_–GO/chi electrode shows efficient and higher current with low potential input in other words, lesser potential was required for oxidation of dopamine to dopamine quinone so that oxidation peak was observed. The results were in correlation with the CV response.

CV responses of modified CP/Fe_3_O_4_–GO/chi electrode in the presence of 50 μM of DA in PBS in the potential range of −0.6 to 0.6 V were recorded and depicted in Fig. S7A in the ESI.† An increase in both oxidation and reduction peaks was observed and its corresponding calibration plot was also obtained (Fig. S7B†), which was linear with an *R*^2^ value of 0.9794 showing the oxidation of DA at the working electrode in a diffusion-controlled process. The correlation between the peak potential (*E*_p_) and the scan rate for a reversible electrochemical reaction can be described by the equation, where *α* represents the charge transfer coefficient, *k* denotes the standard rate constant for the heterogeneous electron transfer, *n* signifies the number of electrons engaged in the reaction, and *E* represents the formal potential. The determination of the *n* value in accordance with the Levron equation involves analyzing the slope of the *E*_p_*vs.* ln(*v*) plot. Additionally, the calculation of the *k* value can be achieved by evaluating the intercept of the plot, provided that the value of *E* is known. The value of *E* can be determined by analyzing the intercept of the plot depicting the relationship between *E*_p_ and *v*.^51^ The rate constants of Fe_3_O_4_–GO and GO were found as 603 and 112 respectively (Fig. S8†). The observed values suggest that the electron transfer phenomenon occurs more strongly in the synthesized nanocomposite, hence accounting for the significant current obtained in the cyclic voltammetry investigations.

### 3.5. Electroanalytical performance of microneedle sensor towards dopamine

**Fig. 5:**
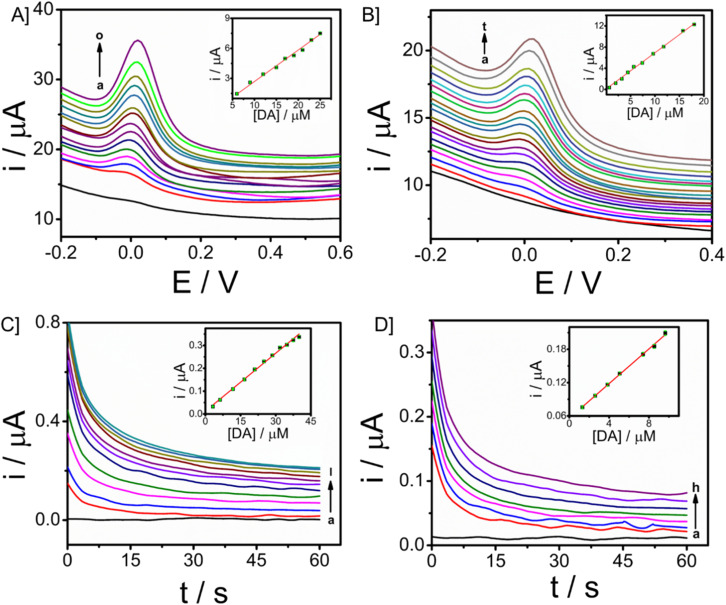
SWV responses of CP/Fe_3_O_4_–GO/chi towards dopamine in 0.1 M PBS (pH-7) with spiking of dopamine in concentrations ranging [A] 3 to 32 μM and [B] 0.66 to 19 μM and corresponding calibration plot between analyte concentration in μM (inset) and *i*/μA. CA responses of CP/Fe_3_O_4_–GO/chi towards dopamine in 0.1 M PBS (pH-7) with an applied potential of 0.03 V *vs.* Ag/AgCl [C] 3 to 40 μM, [D] 1.3 to 10 μM and corresponding calibration plot between dopamine concentration in μM and *i*/μA (inset).

The electroanalytical performance of the microneedle sensor towards DA was evaluated by the orthogonal detection method. SWV and CA techniques were used and corresponding voltammetric and amperometric responses were recorded. SWV responses of the modified electrode towards a long range of DA 3 to 32 μM are shown in Fig. 5A and the linear calibration plot is provided in the inset, from which the sensitivity was found to be 0.31457 μA μM^−1^ (*R*^2^ = 0.94585). Since the response obtained was good, the concentration of DA ranged from 0.66 to 19 μM, short-range data was also obtained (Fig. 5B). From the calibration plot given in the inset, the sensitivity was known as 0.68494 μA μM^−1^ (*R*^2^ = 0.98669) and the limit of detection is 90 nM (3σ method). In both cases, the peak current was obtained at 0.03 V. There is an increase in peak current with an increase in the concentration of DA. From this, it is evident that CP/Fe_3_O_4_–GO/chi can be used to detect dopamine and the amount of DA can also be determined quantitatively.

The peak current was obtained at a potential of 0.03 V from square wave voltammetry, and this is the applied potential for chronoamperometry *versus* Ag/AgCl reference electrode. CA responses were recorded for a time period of 60 seconds. The long-range responses were recorded for DA concentrations, 3–40 μM as shown in Fig. 5C. The increase in current was distinct and well-defined. A linear plot of analyte concentration against the current is provided in the inset with a sensitivity of 0.00845 μA μM^−1^ (*R*^2^ = 0.99285). Further, short-range concentrations 1.3–10 μM The responses are given in Fig. 5D, along with the calibration plot (*R*^2^ = 0.9979) which shows sensitivity towards DA to be 0.01561 μA μM^−1^ and limit of detection is 0.6 μM. For the development of a sensor with integrated electronics, amperometric responses will be efficient and the modified microneedle sensor proves to be suitable for developing a biosensor with integrated electronics. DA monitoring has also been done with Differential Pulse Voltammetry (DPV). The sensor was able to monitor DA at a potential of 0.03 V, which is similar to that of the peak potential of SWV (Fig. S9†). An increase in peak potential was observed with an increase in DA concentrations from 9 μM to 17 μM. A linear calibration plot was also obtained with a sensitivity of 0.11547 μA μM^−1^ (*R*^2^ = 0.9818) provided in the inset of Fig. S9.† The DPV has exhibited the limit of detection of 0.13 μM almost similar to the SWV (which is 0.09 μM).

### 3.6. Real-time detection of DA in artificial ISF

**Fig. 6:**
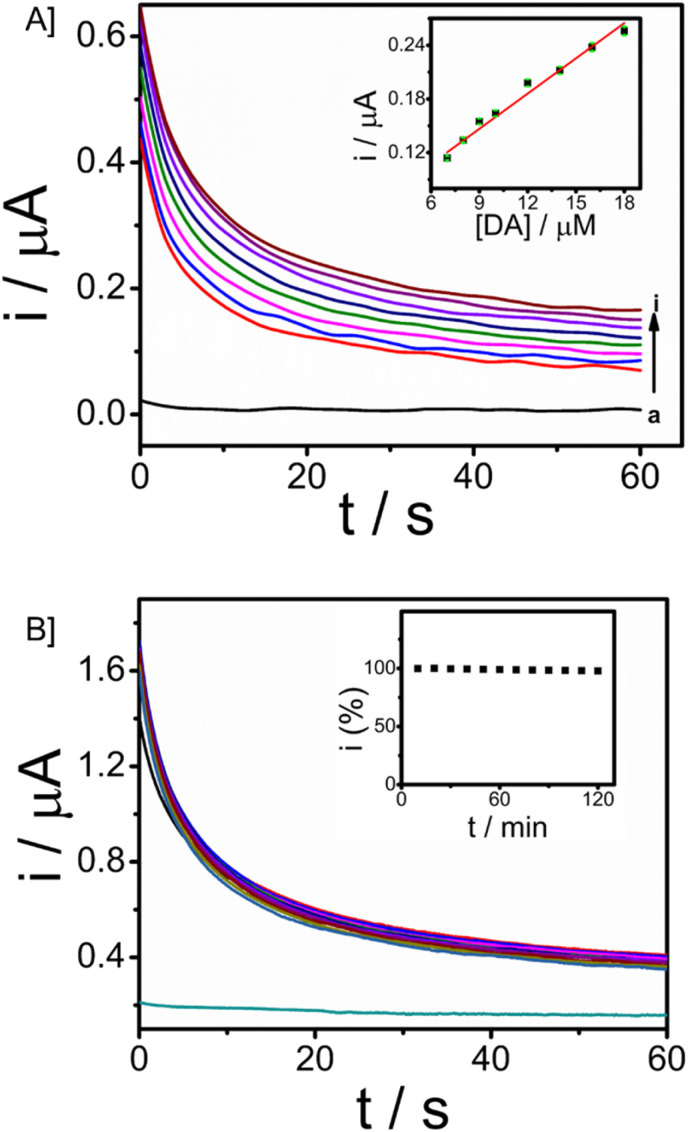
[A] CA responses of microneedle sensor towards dopamine in ISF with an applied potential 0.03 V *vs.* Ag/AgCl reference electrode and Plot between DA concentration and current is provided in the inset. [B] Stability run in chronoamperometry with input potential of 0.03 V *vs.* Ag/AgCl for 120 minutes with DA concentration as 50 μM and corresponding percentage decrease with respect to time (inset).

The minimally invasive nature of microneedle sensors demands the stability of modified electrodes and the detection of dopamine in ISF. For real-time applicability, a study in ISF is essential since ISF carries several analytes, physiological ions and proteins. Artificial ISF was developed for the monitoring of DA, which also contains Bovine serum albumin protein (4 mg ml^−1^). Analysis for the detection of dopamine in ISF was carried out using chronoamperometry. The concentration of dopamine was increased from 7 μM to 18 μM with an applied working potential given as 0.03 V *vs.* Ag/AgCl sampled for 60 seconds. Well-defined amperograms were obtained as the concentration of the analyte was increased. The calibration plot of dopamine in ISF was displayed in Fig. 6A (*R*^2^ = 0.97705), and the response was observed with a sensitivity of 0.01528 μA μM^−1^. From the ISF studies, it was revealed that the microneedle sensor can effectively detect the analyte in ISF and hence can be extended to *in vivo* analysis.

The operational stability of the microneedle sensor is vital for continuous monitoring of dopamine as well as other drug monitoring. Chronoamperometry in artificial ISF with 50 μM of the analyte dopamine in artificial ISF was done to assess the stability, with an input potential of 0.03 V *vs.* Ag/AgCl. The stability analysis was carried out for 120 minutes with a time interval of 3 minutes (10 responses are provided in the inset) and responses were recorded for a period of 60 seconds. Fig. 6B gives the stability of the microneedle sensor in ISF. The system expresses good operational stability and hence can be used for continuous monitoring applications. This stability is attributed to chitosan and the Nafion layer. Chitosan facilitates the formation of a homogeneous transducer layer on the surface, this protects the nature of Fe_3_O_4_–GO nanoparticles over the working electrode. Further Nafion layer is provided, the presence of such a layer is of significant importance in preserving the antifouling characteristics of a wearable sensor.

### 3.7. Skin mimicking gel

**Fig. 7:**
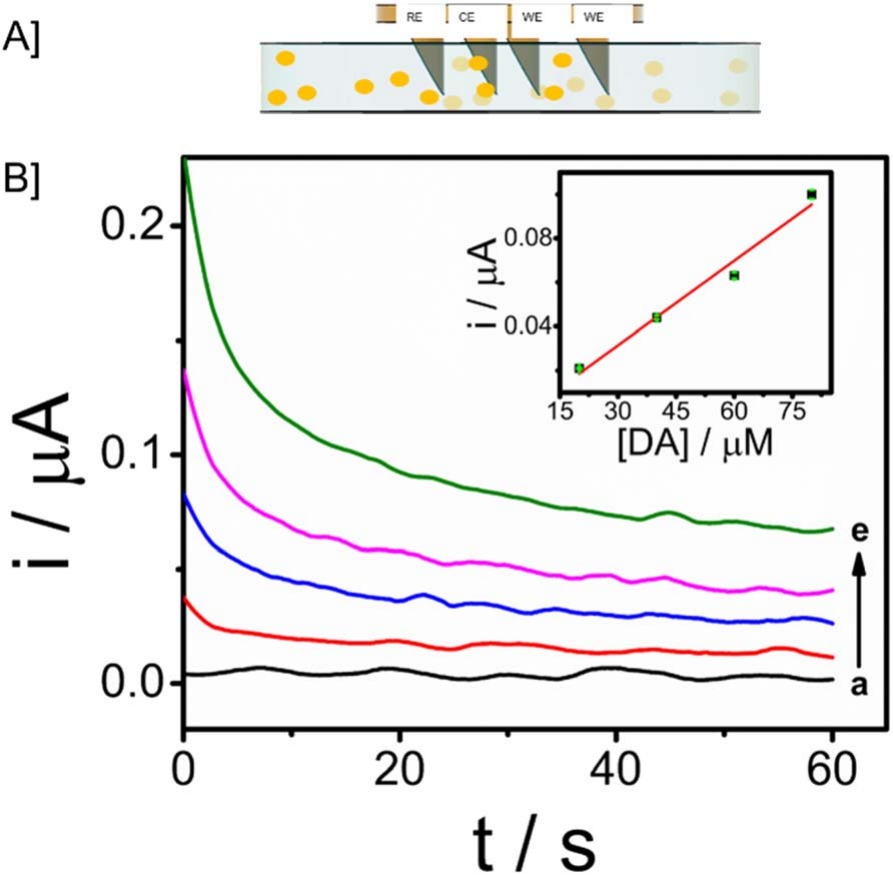
[A] Schematic representation of real-time dopamine monitoring using the microneedle penetrated through the skin-mimicking gel and [B] CA responses of modified electrode towards dopamine in skin-mimicking gel with input potential of 0.03 V *vs.* Ag/AgCl and inset represents the calibration plot between dopamine concentration in μM and *i*/μA.

In order to extend the applicability of this microneedle sensor for monitoring patients in real-time, the stability and monitoring ability of the modified electrode towards dopamine is evaluated in the dermis-mimicked gel. Different concentrations of analyte, 20 μM, 40 μM, 60 μM and 80 μM were prepared and diffused in the phantom skin mimicking gel prepared. Chronoamperometry responses were recorded by providing a potential of 0.03 V *vs.* Ag/AgCl for 60 seconds. Responses are given in Fig. 7B. An increase in current was observed as the gel with a higher concentration of analyte was recorded and the responses were well distinct. Such favourable responses show that this microneedle array system can be applicable in real-time continuous monitoring of patients with neurological disorders through dopamine level abnormalities.

### 3.8. Selectivity, reproducibility and storage stability of microneedle sensor

**Fig. 8:**
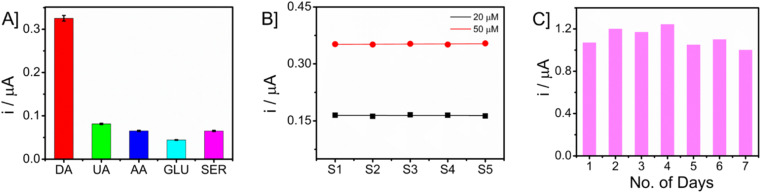
[A] Selectivity of microneedle sensor towards dopamine detection in the presence of potential interferences. [B] The chronoamperometric responses of five distinct modified microneedle sensors were examined for detecting dopamine (DA) at two concentrations (20 and 50 μM). [C] CA responses of microneedle sensor in ambient conditions for monitoring dopamine (50 μM) for a period of 7 days.

The investigation focused on evaluating the selectivity of the proposed sensor when exposed to potential interfering chemicals. The chronoamperometric analysis of glucose (GLU), uric acid (UA), ascorbic acid (AA), and dopamine (DA) was conducted in an artificial interstitial fluid (ISF) medium. The measurements were carried out by taking a 50 μM concentration of dopamine in the presence of a tenfold increased concentration (500 μM) of interfering molecules. The bar graph showing the current responses is presented in Fig. 8A. The Nafion layer covering the electrode surface prevents the nanocomposite from biofouling and behaves as an anti-interferant leading to selective detection of DA. The consideration of reproducibility and long-term storage stability holds significant importance in the development of a sensor. In this study, the repeatability of the measurements was assessed by quantifying the concentrations of DA at two distinct levels (20 and 50 μM) using a set of five separate microneedles. The obtained data are presented in Fig. 8B. The responses of the sensors were found to be similar, and it was observed that the microneedle sensor had a high level of reproducibility. To ensure long-term storage stability, the sensor was maintained at ambient temperature for a duration of seven consecutive days. Subsequently, the sensor's current response to a concentration of 50 μM DA was measured using the technique of amperometry. The obtained responses are shown in Fig. 8C. The sensor that was built exhibited comparable current levels, though with a little reduction.

## 4. Conclusions and future works

A wearable minimal-invasive electrochemical microneedle sensor for monitoring the neurotransmitter dopamine has been demonstrated. The structure and morphology of the developed nanomaterial Fe_3_O_4_–GO were characterized with different analytical techniques. The electrode–electrolyte interface characteristics of the modified electrode were proven to be superior to the unmodified electrode through cyclic voltammetry and square wave voltammetric studies. The electroanalytical performance of microneedles towards dopamine was analyzed in PB solution (pH = 7) using voltammetry and amperometry. Dopamine sensing in artificial ISF gives distinct peaks in chronoamperometry, hence modified electrodes can be used as wearable continuous monitoring sensors. Finally, studies in phantom skin-mimicking gel that mimics human skin also display attractive analytical results. Monitoring with different analytical techniques simultaneously is also possible with different working electrodes. To conclude, the developed microneedle electrochemical sensor can be used for continuous monitoring of dopamine through ISF as a biofluid. The future scope of this work will be focusing on *ex vivo* mouse-skin samples, *in vivo* analysis in both animals and humans, and developing a wireless sensor that can be integrated with minimally invasive microneedles.

## Conflicts of interest

There are no conflicts to declare.

## Supplementary Material

RA-014-D4RA00110A-s001
