# Supplementary material for: Biopolymer-protected graphene-Fe3O4 nanocomposite based wearable microneedle sensor: toward real-time continuous monitoring of dopamine
Source: RSC Adv. 2024 Feb 27;14(10):7131–41. doi: 10.1039/d4ra00110a (PMC10898425; doi:10.1039/d4ra00110a)
Supplement: RA-014-D4RA00110A-s001 [file RA-014-D4RA00110A-s001.pdf]

Electronic Supplementary Material (ESI) for RSC Advances.
This journal is © The Royal Society of Chemistry 2024

SUPPORTING INFORMATION

Biopolymer-Protected Graphene-Fe_3O_4 Nanocomposite Based Wearable Microneedle Sensor: Toward Real-Time Continuous Monitoring of Dopamine.

Keerthanaa M. R. [a], Lakshmi R. Panicker [a], Roger Narayan [b], Yugender Goud Kotagiri [a]*

[a] Department of Chemistry, Indian Institute of Technology Palakkad, Palakkad, Kerala 678 557, India.

[b] Department of Biomedical Engineering, NC State University, Raleigh, NC 27695, USA.

Corresponding Authors Details
Dr. Yugender Goud Kotagiri
Ramanujan Fellow,
Department of Chemistry,
Indian Institute of Technology Palakkad,
Palakkad, Kerala 678 557, India.
E-mail: yugenderkotagiri@iitpkd.ac.in

SUPPORTING INFORMATION

Contents

1. **Synthesis protocol of Fe_3O_4-GO nanocomposite** 2
2. **Stress Analysis on Microneedle** 3
3. **Structural Characterization** 3
   3.1. FTIR Spectrum 3
   3.2. XRD Analysis 4
4. **Electrochemical Optimizations** 4
5. **Electrode-Electrolyte Interface studies-Scan Rate Effect** 5
6. **Electroanalytical Performance of Microneedle Sensor towards Dopamine:** 7

1. Synthesis protocol of Fe_3O_4-GO nanocomposite

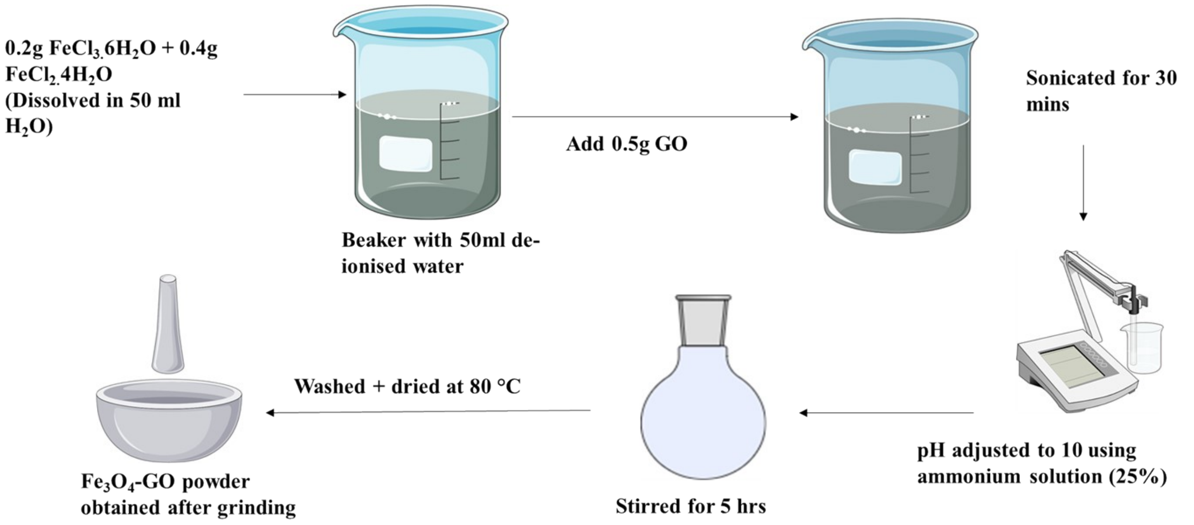

Figure S1: Schematic representation of synthesis protocol of Fe_3O_4-GO composite

2. Stress Analysis on Microneedle

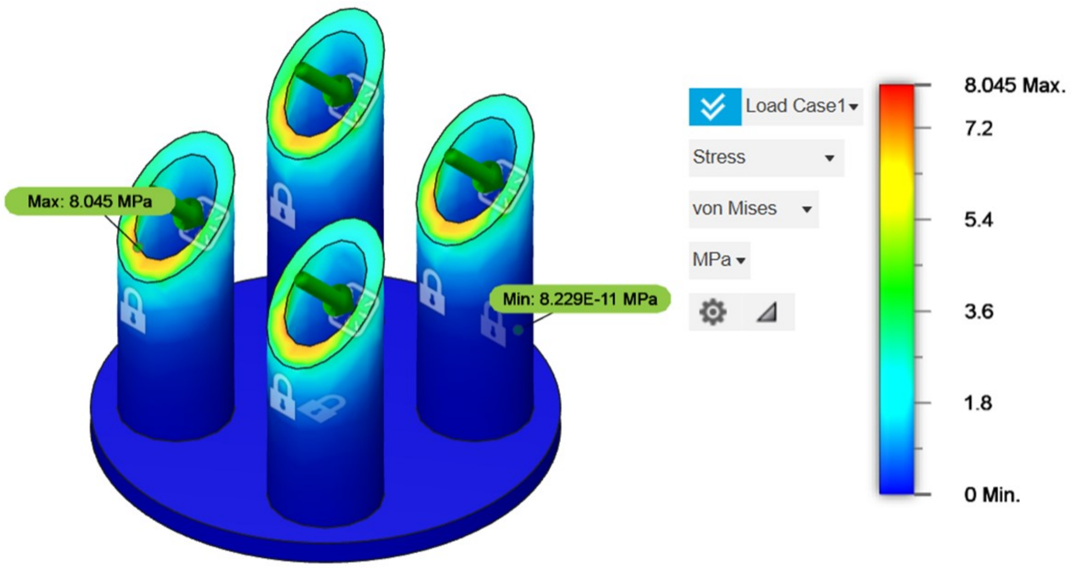

Figure S2: Microneedle insertion force analysis using the Fusion 360 simulation approach.

3. Structural Characterization

3.1. FTIR Spectrum

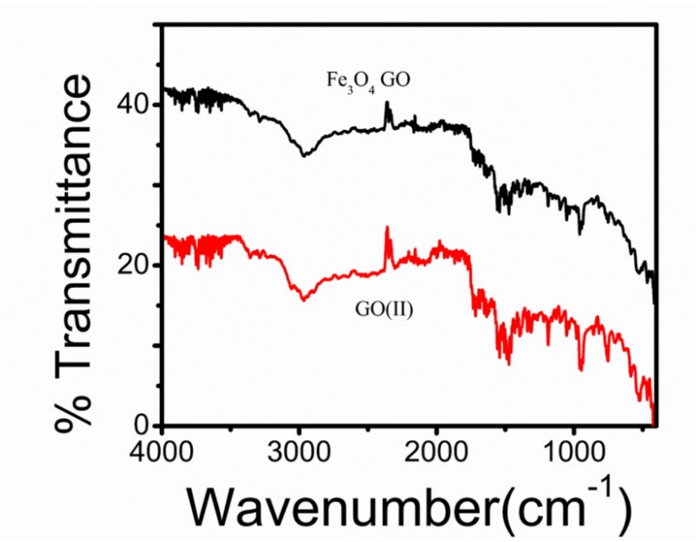

Figure S3: FTIR Spectrum of GO and Fe_3O_4-GO

3.2. XRD Analysis

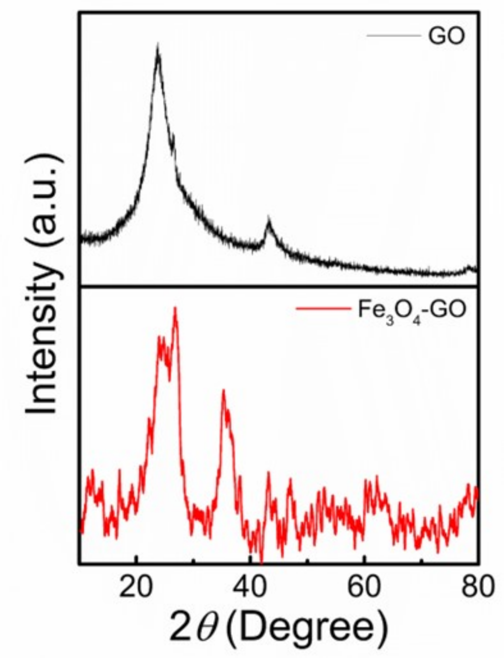

Figure S4: Powder XRD Spectrum of GO and Fe_3O_4-GO

4. Electrochemical Optimizations

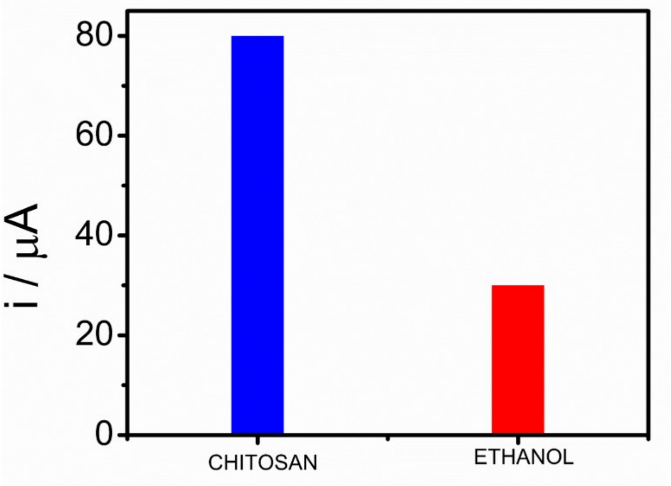

Figure S5: Optimization of Fe_3O_4-GO dissolved in chitosan and ethanol and corresponding current values obtained from cyclic voltammetry with potential applied from -0.2 V to 0.9 V at scan rate of 100 mVs^{-1}.

5. Electrode-Electrolyte Interface studies-Scan Rate Effect

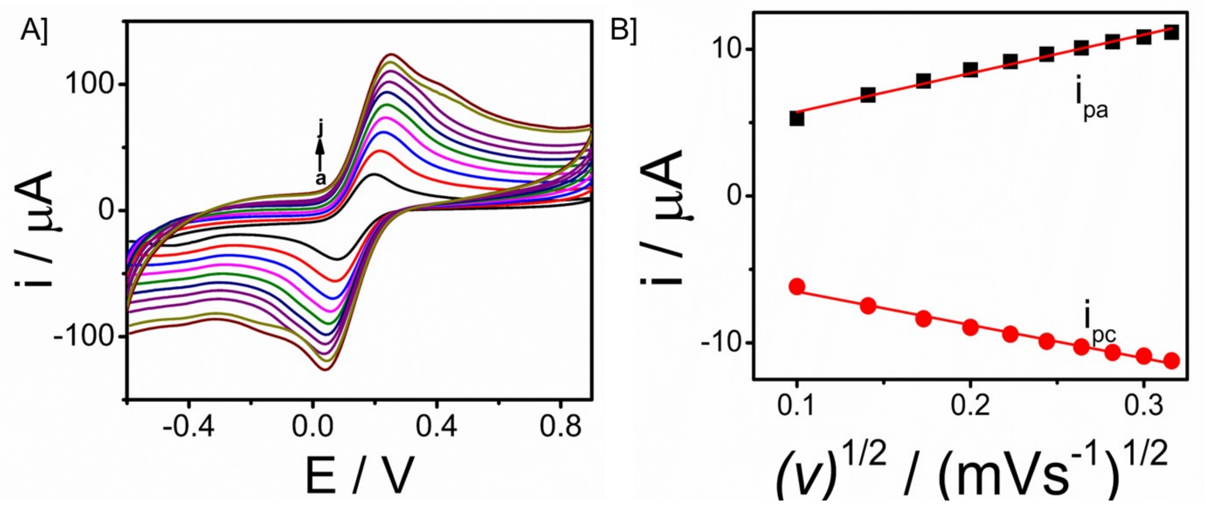

Figure S6: A] Effect of Scan Rate (10-100 mVs^{-1}) on Cyclic Voltammetry of CP/Fe_3O_4-GO/Chi in 10mM $K_3[Fe(CN)_6]$ + 0.1M KCl solution and.B] Plot of anodic (i_{pa}) and cathodic peak (i_{pc}) currents vs. scan rate.

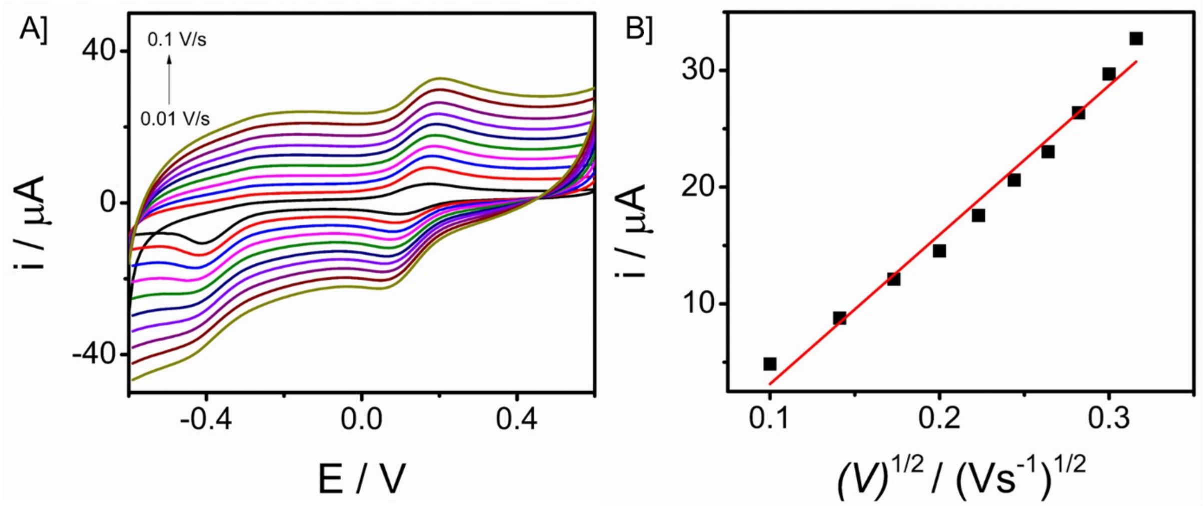

Figure S7: A] CV responses of Scan rate (0.01 V/s to 0.1V/s) Vs Applied potential in presence of 50 μM DA, B] Calibration plot of square root of scan rate Vs peak current

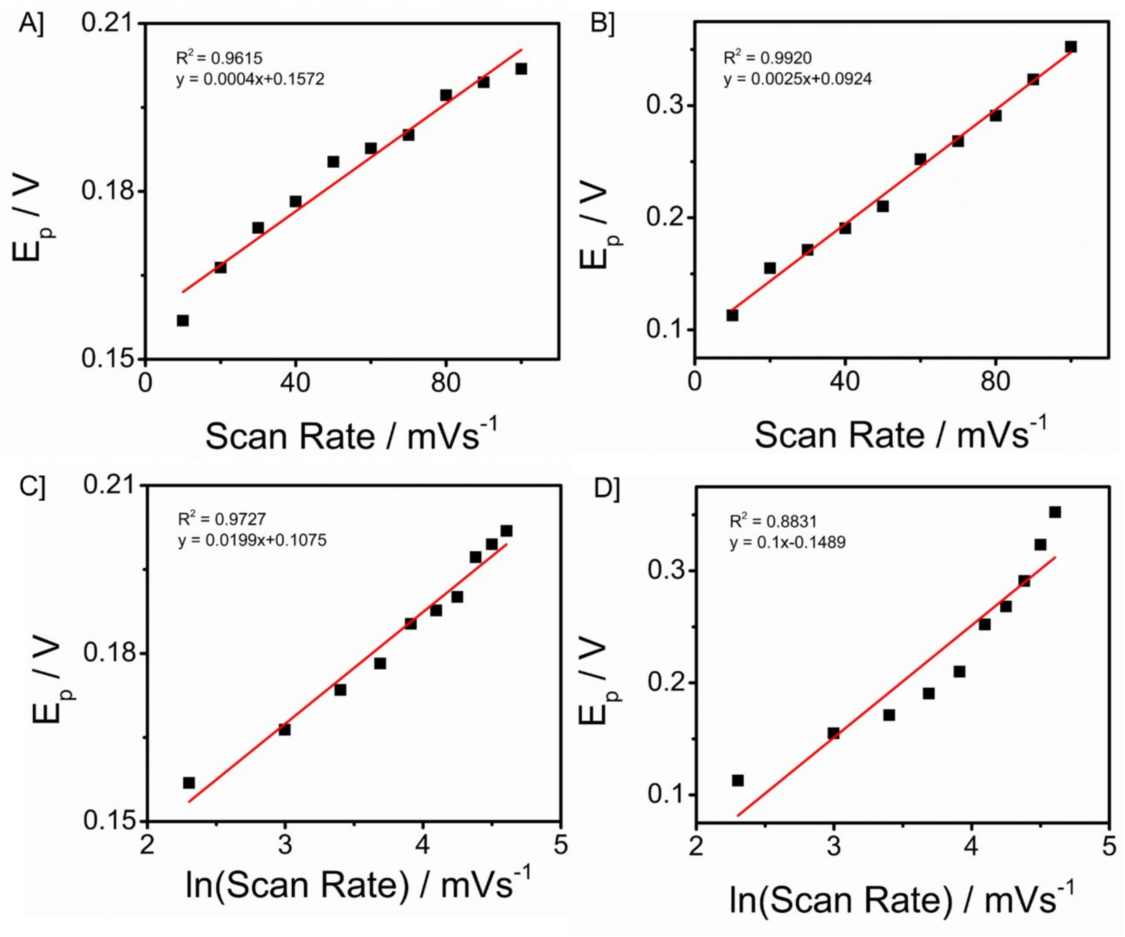

Figure S8: A] Plot of peak potential vs scan rate for 50 µM DA in, C] Calibration plot of peak potential Vs ln of scan rate of PBS CP/Fe_3O_4-GO/Chi electrode, B] Calibration plot of peak potential Vs scan rate and D] Peak potential Vs ln(Scan rate) in PBS containing 50 µM of working electrode modified with GO.

Levron equation – $E_p = E^o + \frac{RT}{\alpha nF}\ln\left[\frac{RTk^o}{\alpha nF}\right] + \frac{RT}{\alpha nF}\ln(v)$

6. Electroanalytical Performance of Microneedle Sensor towards Dopamine:

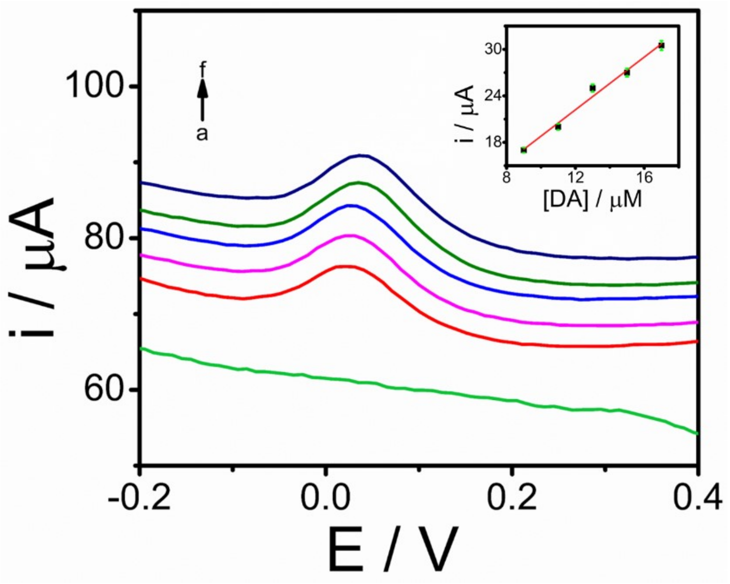

Figure S9: DPV responses of the microneedle sensor towards DA in the concentration range of 9 µM to 17 µM (in 0.1 M PBS) and linear plot between analyte concentration in µM (inset) and i/µA is provided in the inset.
